# Anesthetic management of a patient with esophageal penetration of a tracheal stent: a case report

**DOI:** 10.1186/s40981-016-0068-z

**Published:** 2016-12-03

**Authors:** Yoko Hori, Hiroaki Kishikawa, Atsuhiro Sakamoto

**Affiliations:** Department of Anesthesiology, Nippon Medical School, Sendagi 1-1-5, Bunkyo-ku, Tokyo, 113-8603 Japan

**Keywords:** Tracheal stent, Laryngeal mask

## Abstract

**Background:**

Tracheal stent is a good way to maintain a patent airway in case of stenosis. Although anesthesia techniques for the placement of a stent in the trachea of patients with tracheal stenosis have been reported, the management of general anesthesia in patients with a tracheal stent is not well established.

**Case presentation:**

We report the anesthetic management in the patient with a partly fractured tracheal stent. A 65-year-old man with colon cancer was scheduled for colectomy under general anesthesia. Eight years ago, a tracheal stent was placed because of lung cancer. Preoperative evaluation revealed that a part of the tracheal stent had penetrated the esophagus. We induced general-epidural anesthesia via spontaneous breathing through a laryngeal mask airway to avoid mediastinal emphysema caused by positive pressure ventilation. The patient has been followed up for 2 years without any respiratory complications.

**Conclusion:**

General anesthesia can be safely induced under spontaneous ventilation through a laryngeal mask airway in a patient with a fractured tracheal stent.

## Background

The tracheal stent is a good way to maintain a patent airway in case of stenosis. Although anesthesia techniques for the placement of a stent in the trachea of patients with tracheal stenosis have been reported [1, 2], few papers have discussed the management of general anesthesia in patients with a tracheal stent. Furthermore, this case report also describes a rare event of fracture and penetration of the tracheal stent into the esophagus.

## Case presentation

**Fig. 1:**
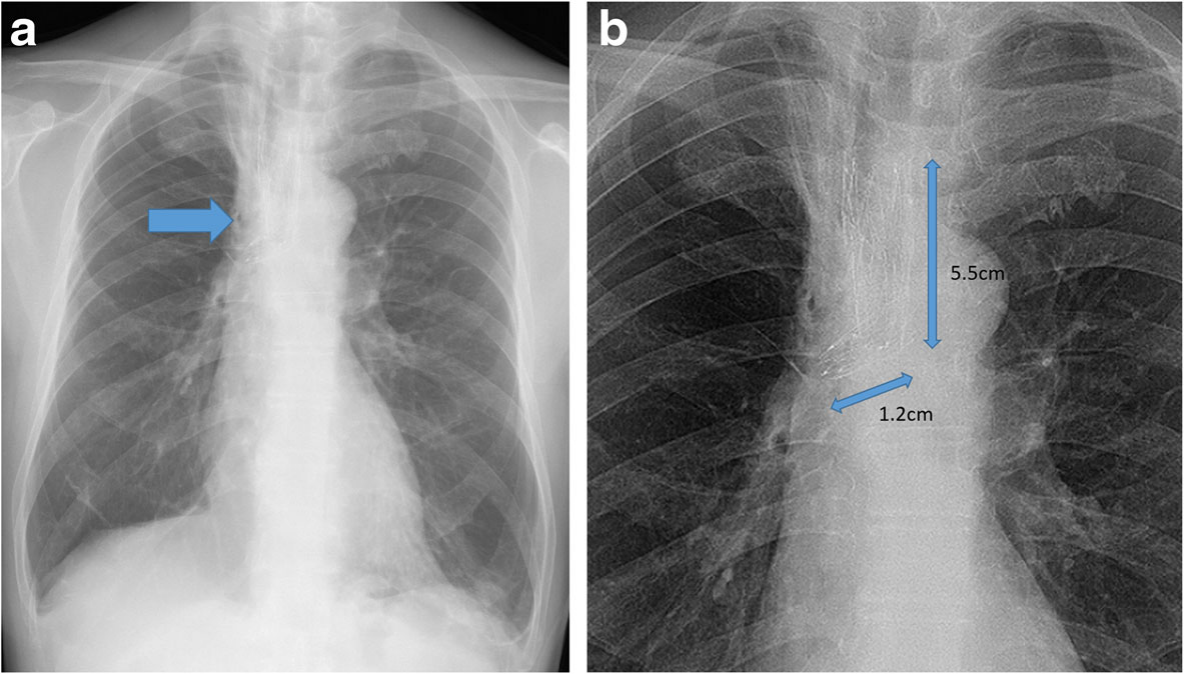
**a** An original chest X-ray is shown. **b** An enlarged X-ray with a high contrast shows the tracheal stent in place between the main bronchus and the main right bronchus

**Fig. 2:**
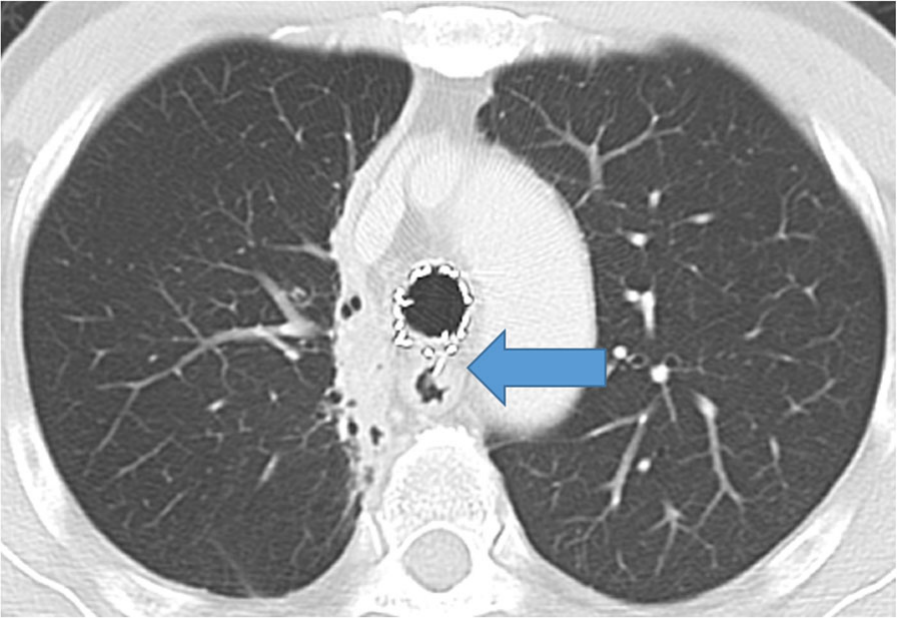
Preoperative chest computed tomography (CT) scan shows that a part of the broken tracheal stent has penetrated into the esophagus

**Fig. 3:**
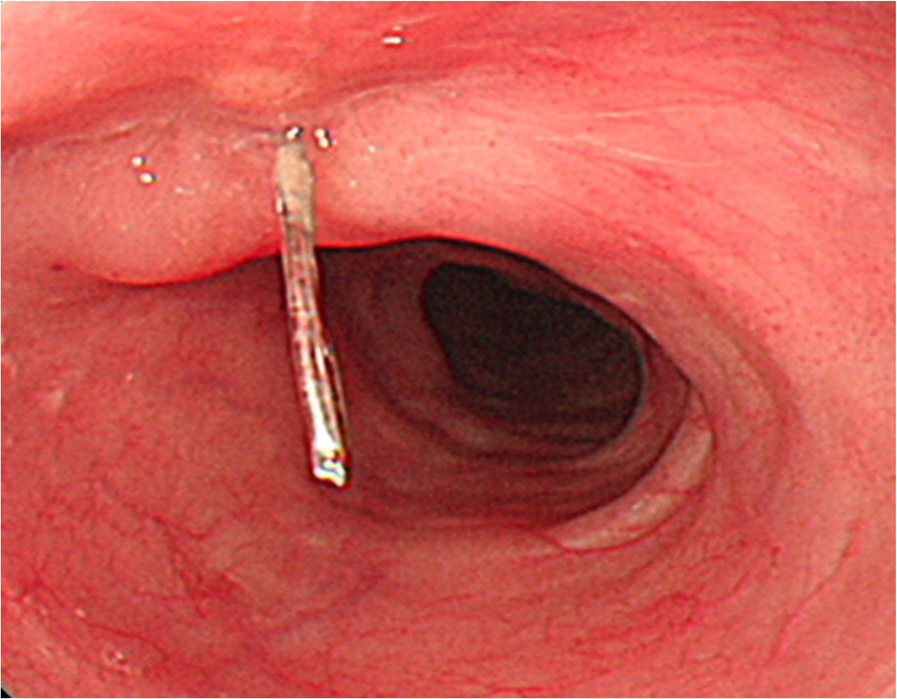
An endoscopic examination of the upper digestive tract shows part of the tracheal stent in the esophagus

A 65-year-old man (height 155 cm, weight 47 kg) was diagnosed with rectal cancer and was scheduled to undergo open colectomy. He had a medical history of limited-disease small cell lung cancer with tracheal stenosis; a tracheal stent procedure had been performed 8 years ago (Fig. 1). During the preoperative assessment, a computed tomography (CT) scan revealed that a part of the tracheal stent placed between the main bronchus and the main right bronchus had penetrated the esophagus (Fig. 2). Endoscopic examination of the upper digestive tract revealed the part of the tracheal stent in the esophagus (Fig. 3). The patient did not experience recurrence of lung cancer or show symptoms of narrowing of the respiratory tract.

We needed to plan the anesthetic management of this patient, especially the intraoperative respiratory management. We planned general-epidural anesthesia via spontaneous breathing because we were concerned that an increase in the airway pressure due to positive pressure ventilation may cause mediastinal emphysema.

An epidural catheter was placed into the epidural space between L3 and L4 and 10 ml of 0.75% ropivacaine was administered to induce analgesia under level Th6. After preoxygenation with 100% oxygen, general anesthesia was induced rapidly using 0.025 mg of fentanyl and 60 mg of propofol, but without the use of a muscle relaxant, which resulted in the loss of consciousness. Spontaneous breathing was maintained and anesthesia was deepened using 4% sevoflurane via laryngeal mask airway (LMA) insertion (ProSeal type, #4) without manual bag mask ventilation. Anesthesia was maintained using 2% sevoflurane and intermittent administration of 0.375% ropivacaine through the epidural catheter.

An open colectomy was performed in the lithotomy position. The head-down position does not allow for the maintenance of adequate ventilation; therefore, a pad along the lower part of the back to straighten the lower abdomen above the pelvis was used for support during the operation. We did not insert a gastric tube because a penetrating wire was present in the esophagus. During the operation, enough spontaneous breathing was maintained as respiratory frequency was about 16 times and tidal volume was about 250 ml. The operation was completed safely, and the LMA was removed at the end of the procedure without any complication.

### Discussion

In this case, we were hesitant to place a tracheal tube through the fractured tracheal stent. Moreover, endotracheal intubation posed a risk of distal movement of the stent and difficulty with extubation owing to interaction between the material of the stent and the endotracheal tube. Furthermore, controlled positive pressure ventilation may have caused air leakage into mediastinal space through the penetrated part of stent. Hung et al. reported the administrations of general anesthesia in a patient with tracheal stent and suggested that the LMA can help maintain a patent airway without harming the trachea or the stent, thus enabling safe induction of general anesthesia [3]. Davis et al. suggested the use of a supraglottic airway device such as the standard LMA or Proseal™ LMA is the safest option when there is no risk of aspiration as it obviates the need for tracheal intubation [4].

The patient’s airway remains patent after placement of LMA, and the tracheal stent remains untouched, thus reducing tracheal trauma. LMA also helps maintain the patient’s spontaneous respiration. The LMA provides good anesthetic maintenance and airway patency in patients with tracheal stents undergoing operation under general anesthesia.

The complications of airway stenting occurring immediately or a long time after stent deployment include migration, airway obstruction, retention of secretions with airway obstruction, cough, infection, sputum retention, granulation of tissue at the proximal or distal end of the stent, metal fatigue, corruption, or respiratory infections [4]. Stent fracture is an uncommon complication, but metal stent fracture had been reported [5]. Zakaluzny et al. discussed that stent breakage requires urgent removal of the stent to minimize the risk of damage to the upper airway, stent collapse, or subsequent distal fragmentation of the metallic pieces that could cause inflammatory lung disease [5]. In our case, we retained the tracheal stent because it was composed of metal. Although a metal stent can be relatively safely detained even for advanced stenosis, withdrawal after long-term detention is difficult because of the stent lumen infiltration. Compared with silicon stents, the prolonged use of metal stents is observed less often because their removal is more difficult. Lunn et al. reported serious complications such as airway obstruction, tracheal rapture, and death during metal stent removal [6, 7]. It is important to carefully consider the risk of removal of metal stent and the necessity.

The patient has been followed up for 2 years with no respiratory complication.

## Conclusions

General anesthesia can be safely induced under spontaneous ventilation through a laryngeal mask airway in a patient with a fractured tracheal stent.

## Abbreviations

CT: Computed tomography
LMA: Laryngeal mask airway

## References

[1] Kaneko T, Itani M, Komasawa N, Ueki R, Tatara T (2012). Anesthesia for tracheal metal stent management utilizing venovenous extracorporeal life support. Masui.

[2] Mieda H, Nagano Y, Iwasaki E, Oishi Y, Sasai T, Shin Y (2012). Two cases of airway stent placement to treat tracheal and bronchial fistula using general anesthesia under spontaneous respiration. Masui.

[3] Hung W-T, Liao S-M, Su J-M (2004). Laryngeal mask airway in patients with tracheal stents who are undergoing non-airway related interventions: report of three cases. J Clin Anesth.

[4] Davis N, Madden BP, Sheth A, Crerar-Gilbert AJ (2006). Airway management of patients with tracheobronchial stents. Br J Anaesth.

[5] Zakaluzny SA, Lane DJ, Mair EA (2003). Complications of tracheobronchial airway stents. Otolaryngol Head Neck Surg.

[6] Murthy SC, Gildea TR, Mehta AC (2004). Removal of self-expandable metallic stents: is it possible?. Semin Respir Crit Care Med.

[7] Lunn W, Feller-Kopman D, Wahidi M (2005). Endoscopic removal of metallic airway stents. Chest.

